# Primary Cells from a *CD46*-Edited Bovine Heifer Have Reduced BVDV Susceptibility Despite Viral Adaptation to Heparan Sulfate

**DOI:** 10.3390/v17050634

**Published:** 2025-04-28

**Authors:** Alexandria C. Krueger, Brian L. Vander Ley, Michael P. Heaton, Tad S. Sonstegard, Aspen M. Workman

**Affiliations:** 1 US Meat Animal Research Center, USDA, Agricultural Research Service, Clay Center, NE 68933, USA; alexandria.krueger@usda.gov (A.C.K.); mike.heaton@usda.gov (M.P.H.); 2 Great Plains Veterinary Educational Center, University of Nebraska-Lincoln, Clay Center, NE 68933, USA; bvanderley2@unl.edu; 3 Acceligen Inc., Eagan, MN 55121, USA; tad@acceligen.com

**Keywords:** bovine viral diarrhea virus, BVDV, cattle, CD46, gene editing, heparan sulfate, MDBK

## Abstract

A precision genome edit in the bovine *CD46* gene (A_82_LPTFS_87_) dramatically reduced bovine viral diarrhea virus (BVDV) susceptibility in a cloned heifer. However, pathogen evolution threatens the long-term efficacy of such interventions. Here, our aim is two-fold: first, to determine whether BVDV can adapt in vitro to use the edited CD46 receptor to infect Madin–Darby bovine kidney (MDBK) cells, and second, to evaluate the ex vivo infectivity of culture-adapted viruses in cells from the *CD46*-edited heifer. Serial passage of BVDV on *CD46*-edited MDBK cells selected for virus variants capable of CD46-independent infection. Virus genome sequencing revealed mutations in the viral E^RNS^ gene predicted to enhance HS-mediated entry. HS adaptation was confirmed by inhibiting virus infection with heparin or Heparinase I/III treatment. A naturally occurring HS-adapted field isolate from a persistently infected calf showed similar results. However, when tested on primary cells from the *CD46*-edited heifer, HS-adapted viruses showed reduced infectivity in skin fibroblasts, monocytes, and lymphocytes in a manner that correlated with HS expression. Thus, although BVDV can adapt to use HS as an alternative entry receptor, HS adaptation does not overcome the protection conferred by the *CD46* edit in all relevant cell types.

## 1. Introduction

Bovine viral diarrhea virus (BVDV) belongs to the family *Flaviviridae* and genus *Pestivirus* and is a highly prevalent and significant pathogen that affects cattle worldwide [1]. Infection of seronegative cattle with BVDV results in a systemic infection associated with gastrointestinal and respiratory diseases, suppression of the immune system, and reproductive failure [2,3]. In the latter, BVDV crosses the placenta and infects the developing fetus, resulting in abortion, congenital malformations, or the birth of persistently infected (PI) calves [4]. Persistent infection develops when the fetus is infected early in gestation and prior to the maturation of the fetal immune system. This early exposure leads to the recognition of viral proteins as “self” antigens, resulting in immunotolerance to the specific BVDV strain, which enables viral replication in all tissues [4]. Consequently, PI calves continuously shed the virus in all bodily secretions, making them the most important source of virus spread in the population [5].

BVDV entry into host cells is mediated by the envelope glycoproteins E^RNS^, E1, and E2. Initial attachment to the cell surface is facilitated by electrostatic interactions between E^RNS^ and negatively charged glycosaminoglycans, such as heparan sulfate (HS), which are thought to concentrate viral particles at the cell surface to enhance subsequent binding to entry receptors [6,7]. Next, BVDV E1-E2 heterodimers bind to the cellular receptor CD46, which triggers virus internalization by clathrin-dependent endocytosis [8,9]. Specifically, CD46 residues E_66_QIV and G_82_QVLAL are required for binding to BVDV E2, facilitating virus entry [8,10,11].

Recently, CRISPR/Cas9-mediated homology-directed repair was used to introduce a 19-nucleotide in-frame substitution within the *CD46* gene. Specifically, the bovine genomic sequences encoding CD46 amino acid residues G_82_QVLAL were replaced with those encoding A_82_LPTFS, resulting in the expression of a novel CD46 receptor variant, herein referred to as the CD46 A_82_LPTFS receptor [12]. The homozygous *CD46* gene edit reduced in vitro BVDV susceptibility to a level comparable to that observed in cells with a complete *CD46* gene deletion (CD46Δ), demonstrating that the CD46 A_82_LPTFS substitution eliminates the virus’ ability to use CD46 to initiate infection. Similarly, a *CD46*-edited heifer calf expressing the CD46 A_82_LPTFS receptor variant had dramatically reduced susceptibility to BVDV as measured by reduced clinical signs and a lack of infection in white blood cells following challenge [12].

Despite the advances in genome editing for disease resistance, the rapid evolution of pathogens threatens the long-term efficacy of such interventions. For example, CRISPR/Cas9-mediated knockouts of CD46 in Madin–Darby bovine kidney (MDBK) cells results in a strong reduction in susceptibility to BVDV and Pestivirus H (Hobi-like pestiviruses) [10,11,13,14]. However, complete pathogen resistance is not achieved, and BVDV can rapidly evolve compensatory mutations in vitro that allow it to more efficiently infect CD46-deficient cells [14]. Specifically, in E^RNS^, a substitution at position 479 from a non-charged glycine (G) to a positively charged arginine (R) (G479R) leads to increased binding to HS that allows for CD46-independent entry, while a C441R substitution improves cell-to-cell spread after initial cell entry [14]. Given the ability of BVDV to switch entry mechanisms to infect MDBK cells lacking CD46, we sought to determine whether recent field isolates of BVDV would adapt to the edited CD46 A_82_LPTFS receptor or use an alternative entry pathway.

Here, our aims were two-fold: (1) to determine whether BVDV can adapt in vitro to circumvent the infection restriction imposed by the CD46 A_82_LPTFS receptor, and (2) to evaluate the infectivity of adapted BVDV isolates in primary cells from the *CD46*-edited heifer. Serial passage of BVDV on *CD46*-edited cells selected for viral variants with increased HS-mediated entry. While these variants readily infected *CD46*-edited MDBK cells, they had reduced infection in primary cells from the *CD46*-edited heifer compared to unedited controls. This difference in infection susceptibility correlated with varying HS expression levels between MDBK cells and primary cells. These results highlight the complex interplay between viral adaptation and host cell context, emphasizing the need to study both in vitro and in vivo systems to fully understand mechanisms of viral escape under distinct selective pressures.

## 2. Materials and Methods

### 2.1. Study Population and Sample Collection

Blood was collected from the *CD46*-edited Gir heifer and unedited control Holstein heifer [12] under the University of Nebraska–Lincoln (UNL) Institutional Animal Care and Use Committee (IACUC) approved Project no. 2111. Blood samples were collected from cattle persistently infected (PI) with BVDV under UNL IACUC approved project no. 1901.

### 2.2. Cell Lines and Viruses

BVDV-free Madin–Darby bovine kidney cells (MDBK; ATCC CCL-22, Lot no. 3752721, passage 113) were obtained from the American Type Culture Collection (ATCC; Rockville, MD, USA). CRISPR/Cas9 gene-editing by homology-directed repair was used previously to generate MDBK cells with a *CD46* gene deletion (CD46Δ) or a 19-nucleotide in-frame substitution in the *CD46* gene [12]. This edit encodes a six amino acid substitution in the BVDV binding domain of the CD46 protein receptor (CD46 A_82_LPTFS). Cells were maintained in Minimum Essential Medium (MEM; Gibco, Grand Island, NY, USA) supplemented with 10% gamma-irradiated fetal bovine serum (FBS; Atlas Biologicals, Fort Collins, CO, USA), 1× antibiotic–antimycotic (Gibco) and 2 mM L-Glutamine (Gibco) under 5% CO_2_ at 37 °C. Primary skin fibroblasts were previously isolated from the *CD46*-edited heifer calf and an unedited control Holstein calf [12]. Primary skin fibroblasts were grown in Dulbecco’s Modified Eagle Medium (DMEM, Corning, catalog no. 10-013-V) supplemented with 15% gamma-irradiated FBS and 1× antibiotic–antimycotic.

BVDV is classified into two species, Pestivirus A (formerly BVDV-1) and Pestivirus B (formerly BVDV-2), according to the International Committee on Taxonomy of Viruses [1]. This classification reflects the current understanding of BVDV’s genetic diversity and taxonomic structure within the Flaviviridae family. Herein, for continuity with the literature on BVDV genotyping, these viruses are termed BVDV-1 and BVDV-2. BVDV isolates were obtained from serum samples collected from calves persistently infected with BVDV (BVDV-PI). Serum was collected in 2021 [12] and 2022 (this study) from beef cattle privately owned by a commercial stocker operation in Missouri, USA. Blood samples were collected from cattle via jugular venipuncture. Serum was promptly separated from the cellular fraction by centrifugation at 1600× *g* for 15 min at 4 °C and serum was stored at −80 °C in 1 mL aliquots until use. The virus was isolated from serum and propagated for three passages on MDBK cells to create virus stocks for infection studies.

Titers were estimated for the non-cytopathic BVDV isolates using two methods. In the first method, virus infection efficiency was normalized between isolates through the use of unedited MDBK cells and the inoculation of two-fold serial dilutions onto the cells. The proportion of cells infected at 20 h post-infection was quantified by flow cytometry using an anti-BVDV E2 antibody as described below in Section 2.8. These approximate titers in which 80% of cells were detected as infected were used as the virus volume needed for a multiplicity of infection (MOI) = 1 in subsequent studies comparing virus infections between edited and unedited cells. In the second method, virus infection efficiency was evaluated based on viral RNA abundance, and the titer was estimated by RT-qPCR with log10 dilutions of the viral samples. Viral RNA was quantified by RT-qPCR with a BVDV-specific primer/probe set [15] as previously described [11]. A standard curve was made by plotting RT-qPCR Ct values against log10 dilutions of the NADL virus (ATCC VR-534™) with a known infectious titer. Linear regression analysis was performed to create a standard for estimating the approximate titer of virus stocks. Each section describes which method was used for determining appropriate virus amounts for each study.

### 2.3. Passaging Virus Isolates on CD46-Edited MDBK Cells

Three noncytopathic BVDV strains (PI-90-2021, PI-91-2021, and PI-92-2021) were previously isolated from PI calves and shown to be unable to efficiently infect MDBK cells lacking CD46 (MDBK-CD46Δ) or those expressing the gene-edited CD46 A_82_LPTFS protein receptor (MDBK-CD46 A_82_LPTFS) [12]. These three virus isolates were each passed in series for 11 rounds on three separate cell lines: MDBK, MDBK-CD46Δ, and MDBK-CD46 A_82_LPTFS. For each round of passage, cells were seeded in 24-well plates one day prior to infection. Before the initial passage, cell culture medium was removed and cells (70–80% confluent) were inoculated with BVDV input samples at a MOI of 1. Virus adsorption and entry were allowed to proceed at 37 °C for 2 h and the unbound virus was removed by washing the cells two times with phosphate-buffered saline (PBS). Cells were incubated with MEM containing 5% horse serum (HS; ATCC), 1× antibiotic–antimycotic, and 2 mM L-Glutamine for 72 h. At 72 h post-infection, cells were frozen at −80 °C. For the first passage, the cells were frozen and thawed two times and 350 µL of the harvested 500 µL of crude cell extract was inoculated onto freshly seeded cells. For passages 2–11, clarified cell culture supernatant from the previous passage was used as the inoculum.

### 2.4. Whole Genome Sequencing, Assembly, and Comparisons of BVDV Isolates

Either field-collected serum samples or clarified cell culture supernatants were treated with RNase and DNase as previously described [16] to degrade host nucleic acid. Total RNA was then isolated using a phenol and guanidine isothiocyanate solution according to the manufacturer’s instructions (Trizol LS, Thermo Fisher Scientific, Waltham, MA, USA). One-hundred nanograms of RNA was used as the input material for an RNA library preparation kit (TrueSeq Stranded mRNA kit, Illumina, San Diego, CA, USA). RNA libraries were constructed as specified by the manufacturer’s protocol without the initial step of poly(A) selection on oligo(dT) beads to allow the sequencing of viral genomes and genome fragments lacking poly(A) tails. RNA libraries were sequenced on a desktop sequencer (Illumina NextSeq 2000, San Diego, CA, USA) with a 300-cycle kit to generate 2 × 151-bp paired-end reads. Raw sequence reads were processed using commercial software (Geneious Prime (version 2022.2.2); Biomatters, Auckland, New Zealand). Adapters were trimmed from raw sequence reads using bbduk (version 38.84) as implemented in Geneious. Trimmed reads were de novo assembled using the SPAdes assembler for metagenomic datasets (v.3.15.2). Trimmed reads were then mapped to the appropriate de novo assembled BVDV genome using the Geneious assembler to extract the consensus sequence. The de novo-assembled and consensus genomes were aligned using Geneious alignment and manually inspected for differences at the nucleotide and amino acid levels. For the E^RNS^ gene region, nucleotide frequencies were examined and nonsynonymous nucleotide mutations in greater than 0.5% of the mapped reads were reported. BVDV consensus genomes were submitted to Genbank under accession numbers PQ613778-PQ613798.

### 2.5. Serum Virus Infections of CD46-Edited MDBK Cells

Whole blood was collected from cattle persistently infected with BVDV, and serum was separated as described above in Section 2.2. Cells were seeded 1 day prior to infection in 24-well plates in MEM supplemented with 10% gamma-irradiated FBS, 1× antibiotic–antimycotic, and 2 mM L-Glutamine under 5% CO_2_ at 37 °C. Prior to infection, the cell culture medium was removed and cells washed with MEM containing no FBS three times. Then, cells were inoculated with a 200 μL volume containing 100 µL BVDV serum and 100 µL MEM, 1× antibiotic–antimycotic and 2 mM L-Glutamine. Virus adsorption and entry were allowed to proceed for 2 h at 37 °C, and unbound virus was removed by washing the cells four times with PBS. Cells were incubated with MEM supplemented with 5% horse serum, 1× antibiotic–antimycotic, and 2 mM L-Glutamine for 72 h before total BVDV infection was determined by flow cytometry as described below.

### 2.6. Infection of MDBK Cells with or Without Heparin Pre-Treatment

Cells were seeded one day prior to infection in 24-well plates in MEM supplemented with 10% gamma-irradiated FBS, 1× antibiotic–antimycotic, and 2 mM L-Glutamine. Prior to infection, cell culture medium was removed and cells (70–80% confluent) were washed three times with serum-free MEM prior to inoculation with indicated BVDV isolates at a dilution that infected approximately 80% of the control MDBK cells as determined by flow cytometry. Virus adsorption and entry were allowed to proceed for 2 h at 37 °C and the unbound virus was removed by washing the cells four times with PBS. Cells were incubated with MEM supplemented with 5% horse serum, 1× antibiotic–antimycotic, and 2 mM L-Glutamine for 20 h. BVDV infection was quantified by flow cytometry as described below.

To block heparan sulfate (HS) binding sites on the virus envelope proteins, virus dilutions were pre-incubated with 200 µg/mL heparin (H3149-10KU, Sigma-Aldrich, St. Louis, MO, USA), a HS mimetic [17,18]. Virus was incubated with heparin at 37 °C for 30 min with gentle mixing every 10 min. Virus infections were then conducted as described above.

### 2.7. Heparinase Treatment and Infection of MDBK Cells

MDBK cells were washed three times with PBS and followed by a single wash with Heparinase dilution buffer (PBS containing 0.2% bovine serum albumin, 0.5 mM CaCl_2_, and 0.5 mM MgCl_2_). Cells were then pre-treated with 1 IU Heparinase I/III (Sigma) in a Heparinase dilution buffer for 1 h at 37 °C. Cells were washed twice in Heparinase dilution buffer to remove Heparinase. BVDV was next inoculated at a dilution that infects approximately 60% of the untreated cells at 20 h after infection as determined by flow cytometry. Virus adsorption was allowed to proceed for 30 min at 37 °C. Unbound virus was removed by washing the cells four times with PBS. Cells were incubated with MEM supplemented with 5% horse serum, 1× antibiotic–antimycotic, and 2 mM L-Glutamine for 20 h. BVDV infection was quantified by flow cytometry as described below.

### 2.8. Flow Cytometric Detection of BVDV Antigen

Cells were enzymatically dissociated with a proprietary trypsin-based reagent (TrypLE Express, Gibco, Waltham, MA, USA) and collected by centrifugation for 2 min at 400× *g* at 4 °C. The cells were washed with PBS and fixed with 4% paraformaldehyde (PFA) at room temperature for 12 min. Following fixation, cells were washed with PBS and blocked for 30 min with a blocking and permeabilization buffer (PBS containing 2% bovine serum albumin (BSA) and 0.1% *w*/*v* saponin). Cells were pelleted and resuspended in 100 µL of an antibody dilution buffer (PBS containing 1% BSA and 0.1% *w*/*v* saponin) containing a 1:200 dilution of an anti-BVDV monoclonal antibody (VMRD, Catalog no. 348. Pullman, WA, USA) used for MDBK cells or (DMAB, Catalog no. 28412. Creative Diagnostics, Shirley, NY, USA) used for lymphocytes and incubated for 30 min at room temperature. The cells were washed three times with PBS and resuspended with 100 µL of antibody dilution buffer containing a 1:50 dilution of a fluorescent anti-mouse antibody (CruzFluor™ 488, Santa Cruz Biotechnology, Dallas, TX, USA, Catalog no. sc-516248). Cells were incubated for 30 min in the dark, washed twice with PBS, and analyzed with a flow cytometer (Attune NxT, Thermo Fisher Scientific).

### 2.9. Flow Cytometric Detection of Heparan Sulfate (HS)

For the quantification of HS surface expression, cells were enzymatically dissociated with a proprietary trypsin-based reagent (TrypLE Express), collected by centrifugation for 2 min at 400× *g* at 4 °C, quantified with an automated cell counter (Countess II, Invitrogen, Waltham, MA, USA), and diluted to 2 × 10^5^ cells per tube. Cells were fixed with 4% PFA, washed with PBS, and blocked with 2% BSA-PBS for 30 min at room temperature. A 1:200 dilution of an anti-HS antibody (USBiological, Salem, MA, USA, catalog no. H1890) in 1% BSA-PBS was added to the tubes and incubated for 30 min at room temperature. The cells were washed three times with PBS and resuspended with 1% BSA-PBS containing a 1:2000 dilution of anti-mouse IgM FITC conjugated secondary antibody (AbCam, Cambridge, United Kingdom, catalog no. AB6717). Cells were incubated for 30 min in the dark, washed two times with PBS, and analyzed with a flow cytometer (Attune NxT, Thermo Fisher Scientific).

### 2.10. Infection of Primary Skin Fibroblasts

Cells were infected with various BVDV isolates at a dilution that infects approximately 70% of the unedited fibroblast cells at 20 h after infection as determined by flow cytometry. The same methodology was performed as described above in Section 2.6. Infected cells were quantified by flow cytometry at 20 h post-infection as described above for the detection of BVDV antigen.

### 2.11. Monocyte and Lymphocyte Isolation and Ex Vivo BVDV Challenge

Blood was collected from the *CD46*-edited Gir heifer and the unedited control Holstein heifer via jugular venipuncture into syringes containing EDTA as an anticoagulant. Peripheral blood mononuclear cells (PBMCs) were isolated by density gradient centrifugation with proprietary tubes (SepMate, Stemcell Technologies, Cambridge, MA, USA; ref. [12]). Monocytes were separated from lymphocytes by adherence selection as previously described [12].

Lymphocytes were resuspended at 1.25 × 10^6^ cells per mL RPMI 1640 medium (Cytiva; Marlborough, MA, USA) supplemented with 1× antibiotic–antimycotic (Gibco) and 2.5 × 10^5^ cells (200 µL) were plated in well of a 96-well round-bottom plate. The plate was centrifuged for 2 min at 400× *g* at 4 °C and 170 µL of medium was removed. Lymphocytes were then infected with either (1) an equivalent virus titer based on infectivity in MDBK cells, with the volume brought up to 80 µL with RPMI 1640 medium (average MOI = 3 for all virus pairs), or (2) 80 µL of virus inoculum with various MOI (average MOI = 5 for unadapted viruses and MOI = 27 for adapted viruses) so as to add the maximum amount of each virus isolate to the cells. Lymphocytes were incubated with virus for 2 h at 37 °C to allow virus absorption and then 80 μL of RPMI supplemented with 1× antibiotic–antimycotic and 5% (*v*/*v*) heat-inactivated FBS was added to each well. Lymphocytes were incubated 20 h at 37 °C with 5% CO_2_ and processed for the flow cytometric quantification of BVDV-infected cells as described above in Section 2.8.

Monocytes (3.5 × 10^5^ cells in each well of a 48-well plate) were inoculated with equal titers of BVDV based on input viral RNA concentrations determined by RT-qPCR. Infection was carried out for 2 h at 37 °C with 5% CO_2_ with the gentle rocking of the plates every 15 min. Two-hundred-and-fifty μL of RPMI supplemented with 1× antibiotic–antimycotic and 5% (*v*/*v*) heat-inactivated FBS was then added to each well, and the cells were incubated for 48 h at 37 °C with 5% CO_2_. Input (*t* = 0) samples were also collected and stored at −80°C. Duplicate plates were frozen at 48 hpi and processed for viral RNA detection. Following two freeze–thaw cycles to release viral RNA from the infected cells, RNA was extracted from clarified supernatants using the Qiagen viral RNA spin columns per the manufacturer’s instructions. Viral RNA was quantified by RT-qPCR with a BVDV-specific primer/probe set [15] as previously described [11]. Cycle threshold (Ct) values less than 38 were considered positive. Positive, negative, no-template, and extraction controls were included on each run. The fold increase in viral RNA compared to the input concentration was determined with the delta Ct method and graphed with commercially available software (Prism v6, GraphPad Software; San Diego, CA, USA).

### 2.12. Statistical Analyses

Statistical analyses were performed using GraphPad Prism (version 10.2.3). Infectivity data, derived from at least three independent experiments, were analyzed using a two-way analysis of variance (ANOVA) to assess the main effects of cell status (edited vs. unedited) and virus adaptation (unadapted vs. adapted), as well as their interaction. Where statistically significant main effects or interactions were observed (*p* < 0.05), pairwise comparisons were conducted using Sidak’s multiple comparisons test to determine specific differences between group means.

## 3. Results

### 3.1. Evolution of Three BVDV Field Strains After 11 Passages in CD46-Edited MDBK Cells

**Figure 1:**
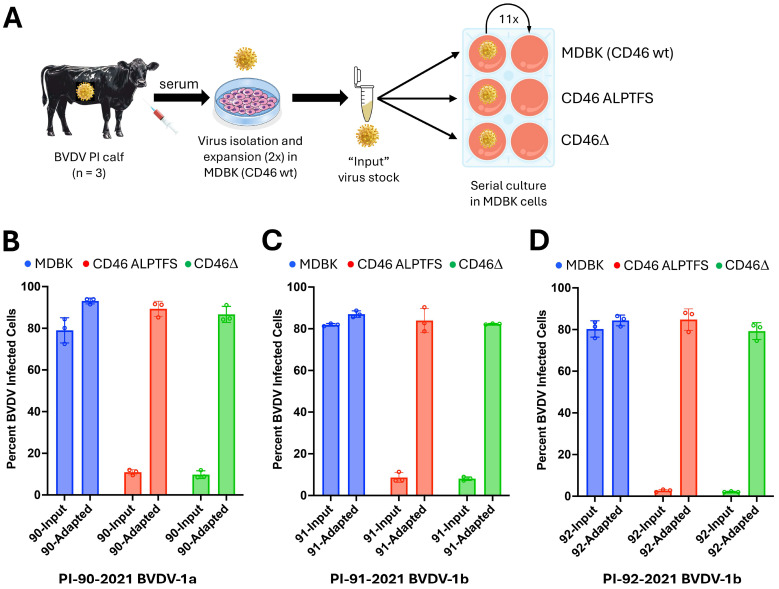
Serial passage and infectivity of viruses adapted to infect *CD46*-edited MDBK cells. (**A**) Serum from BVDV-PI calves (n = 3) was inoculated on MDBK cells to isolate and propagate the virus. These viral stocks (‘input virus’) were subsequently used for serial passage on *CD46*-edited cells. (**B**–**D**) Infectivity of three field strains of BVDV after adaptation to *CD46*-edited MDBK cells. BVDV infection was quantified by flow cytometry at 20 hpi using an anti-BVDV E2 antibody. Results represent the mean ± standard deviation of three independent experiments.

Three noncytopathic field strains of BVDV were serially passaged on three isogenic host cell lines: unedited MDBK cells, MDBK cells expressing the edited CD46 A_82_LPTFS protein receptor, and MDBK cells lacking CD46 (MDBK-CD46Δ) (Figure 1A). Prior to serial passage, input (unadapted) viruses were unable to efficiently infect *CD46*-edited cells. After 11 passages on MDBK-CD46 A_82_LPTFS cells, the adapted viruses infected MDBK, MDBK-CD46 A_82_LPTFS cells, and MDBK-CD46Δ cells with comparable efficiency, indicating the adapted viruses were now capable of using a CD46-independent mechanism of entry (Figure 1B–D).

**Figure 2:**
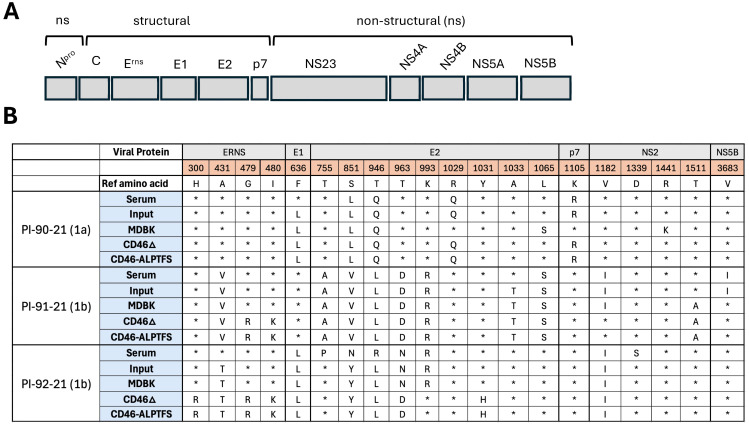
Sequence evolution of in vitro-adapted viruses. (**A**) BVDV genome structure (approximately 12.3 kb). (**B**) Amino acid comparison of sequenced viruses to reference BVDV stain NADL (genotype 1a) GenBank NC_001461. Asterisk (*) indicates the amino acid is the same as the reference sequence in that position. Virus sequences were generated directly from serum (serum), after initial isolation and two expansion passages on MDBK cells (input), or after 11 additional passages of the input virus on MDBK, MDBK-CD46Δ, or MDBK-CD46 A_82_LPTFS cells.

BVDV genome sequences were obtained from serum and input virus stocks and isolates serially passaged on *CD46*-edited MDBK cells. Viral sequence analyses of these samples identified several nucleotide changes associated with serial passage (Supplemental Table S1). Nonsynonymous mutations were predominant in the viral surface glycoproteins (Figure 2). Of these, the substitution of glycine (G) to arginine (R) at position 479 (G479R) in E^RNS^ is a known HS-adaptive substitution [17,19] and a strong candidate for the observed increased infectivity in *CD46*-edited cells for two of the adapted isolates (Table 1, PI-91-2021 and PI-92-2021). Importantly, similar mutations were found whether the viruses were passaged on MDBK-CD46Δ or MDBK-CD46 A_82_LPTFS cells, indicating that the same adaptation mechanisms were likely occurring with both cell lines for these two isolates.

However, the consensus sequence of the third adapted isolate (PI-90-2021) revealed no acquired substitutions in its surface glycoproteins that were specific to passaging on the *CD46*-edited cells. Consequently, quasispecies analysis, focusing on the E^RNS^ gene, was conducted to identify low-frequency (<50%) mutations potentially contributing to the phenotype. While 19 nonsynonymous mutations were identified in this adapted isolate at a frequency greater than 0.5%, only 5 occurred in greater than 2% of the virus population (Supplemental Table S2). Notably, a methionine (M) to arginine (R) substitution at position 421 (M421R) was detected in 34.9% of the nucleotide reads for the MDBK-CD46 A_82_LPTFS cell-adapted virus and is a possible candidate for the altered phenotype, given the substitution of a positive charge that is common in HS-adaptive motifs.

### 3.2. Disruption of HS-Mediated Entry Inhibits Adapted Virus Infection of MDBK-CD46 A_82_LPTFS Cells

**Figure 3:**
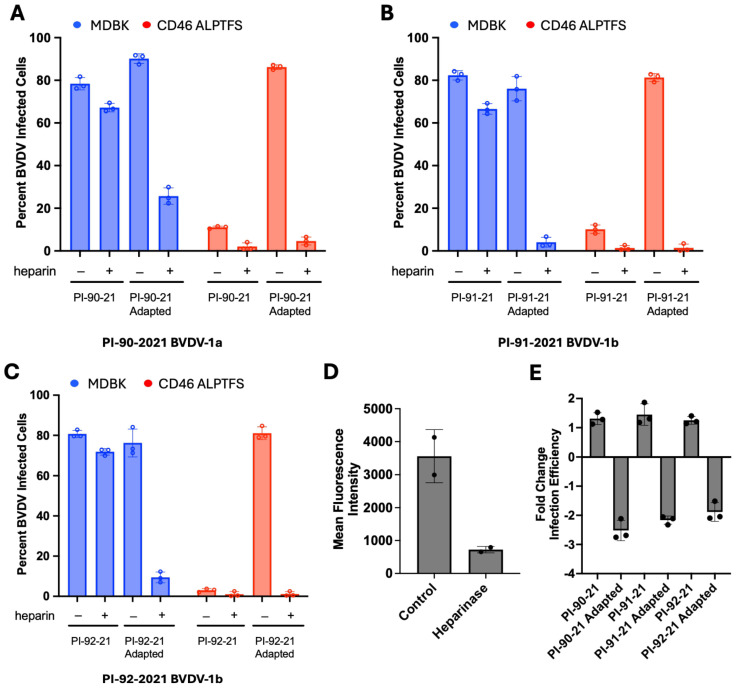
Disruption of HS-mediated virus entry inhibits adapted virus infection of CD46 A_82_LPTFS_87_ cells. (**A**–**C**) MDBK and MDBK-CD46 A_82_LPTFS cells were inoculated with unadapted input virus isolates or p11-adapted virus isolates from MDBK-CD46 A_82_LPTFS cells with or without pre-treatment with heparin. BVDV infection was quantified by flow cytometry at 20 hpi. Results represent the mean ± standard deviation of three independent experiments. (**D**) MDBK cells were treated or not with Heparinase I/III and harvested at the end of virus infection to examine heparan sulfate (HS) abundance available during infection. HS abundance was quantified by flow cytometry. Results represent the mean ± standard deviation of two independent experiments. (**E**) MDBK cells were treated or not with Heparinase I/III and inoculated with unadapted input virus isolates or p11-adapted virus isolates from MDBK-CD46 A_82_LPTFS cells. BVDV infection was quantified by flow cytometry at 20 hpi. Results represent the mean of the fold change in infection between control and Heparinase-treated cells ± standard deviation of three independent experiments.

To test whether the adapted BVDV isolates were using a HS-dependent entry mechanism to infect *CD46*-edited MDBK cells, infection was measured with or without pre-treatment with heparin, an analog that mimics heparan sulfate. Pre-treating the three unadapted viral isolates with heparin reduced the already low level of infectivity observed on MDBK-CD46 A_82_LPTFS cells, indicating that a minor population of the viral quasispecies utilized a HS-dependent entry pathway in the unadapted virus stock (Figure 3A–C). The frequency of the viral quasispecies that carries the G479R substitution in the unadapted input virus samples, however, is less than 0.5% compared to more than 90.0% for two of the adapted virus populations (Table 1). Similarly, heparin pre-treatment of the adapted viruses significantly blocked infection of MDBK-CD46 A_82_LPTFS cells (Figure 3A–C). Surprisingly, heparin pre-treatment of the adapted viruses also substantially reduced infection in the unedited MDBK cells. In contrast, the unadapted input viruses were only slightly reduced in their ability to infect MDBK cells following heparin pre-treatment.

In a complementary method, Heparinase I/III was used to remove cell surface heparan sulfate from cells prior to infection. Heparinase pre-treatment of cells reduced the mean fluorescence intensity (MFI) of HS to approximately 20% of the control treatment (Figure 3D). This degree of HS removal from the cell surface was sufficient to decrease adaptive virus infection by approximately two-fold. In contrast, HS removal resulted in a slight increase in the infection of unadapted viruses (Figure 3E). Together, these results suggest that the adapted viruses are using HS to initiate infection, thereby overcoming the restriction observed in MDBK-CD46 A_82_LPTFS cells. Furthermore, they suggest viral adaptation to HS may impair the viruses’ ability to use CD46 to enter unedited cells.

### 3.3. A Virus Isolate from PI Yearling Calf Infects CD46-Edited MDBK Cells

**Figure 4:**
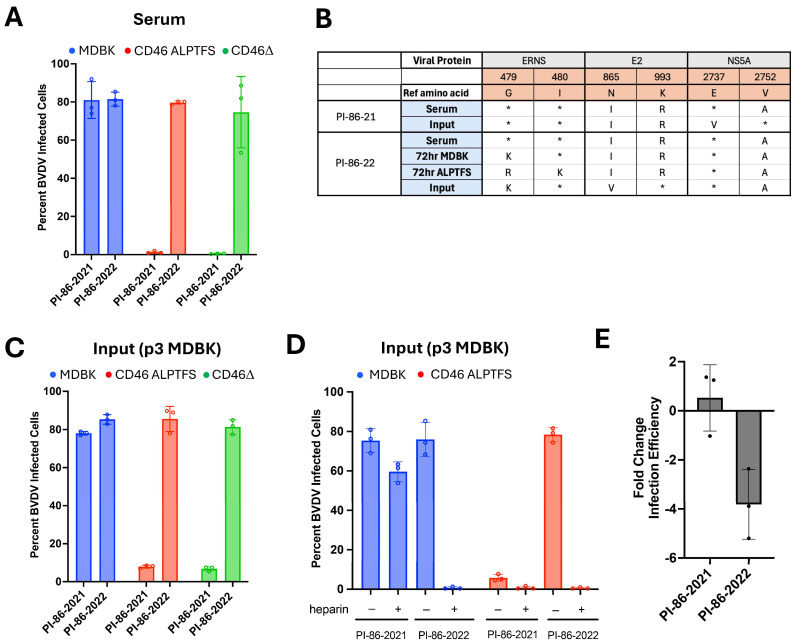
A heparan sulfate-adapted virus arose in a yearling calf persistently infected with BVDV. (**A**) Serum was collected from the same BVDV-PI calf in Nov of 2021 (PI-86-2021) and Nov 2022 (PI-86-2022). Serum was inoculated on MDBK, MDBK-CD46Δ, and MDBK-CD46 A_82_LPTFS cells. BVDV infection was quantified by flow cytometry at 72 hpi. Results represent the mean ± standard deviation of three independent experiments. (**B**) Serum was inoculated on MDBK cells to isolate and propagate the virus. Serum samples, virus samples from a single round of infection (72 h) on MDBK and MDBK-CD46 A_82_LPTFS cells, and virus stocks (p3 on MDBK, ‘input virus’) were sequenced. Nonsynonymous mutations are organized by virus protein. The asterisk (*) indicates the amino acid is the same as the reference sequence in that position. (**C**) Input viruses were inoculated on MDBK, MDBK-CD46Δ, and MDBK-CD46 A_82_LPTFS cells. BVDV infection was quantified by flow cytometry at 20 hpi. Results represent the mean ± standard deviation of three independent experiments. (**D**) Input viruses (p3) were pre-treated with or without heparin and infection was quantified at 20 hpi by flow cytometry. Results represent the mean ± standard deviation of three independent experiments. (**E**) MDBK cells were untreated or treated with Heparinase and inoculated with p3 input viruses. BVDV infection was quantified by flow cytometry at 20 hpi. Results represent the mean of the fold change in infection between control and Heparinase-treated cells ± standard deviation of three independent experiments.

As part of a routine BVDV surveillance, two BVDV strains (PI-86-2021 and PI-86-2022) were isolated from the same BVDV PI calf, with approximately one year between the collection dates. The BVDV strain in the first serum sample collected from this animal, PI-86-2021 was unable to efficiently infect MDBK-CD46Δ and MDBK-CD46 A_82_LPTFS cells. In contrast, the BVDV strain in the second serum sample, PI-86-2022, was able to efficiently infect both the MDBK-CD46Δ and MDBK-CD46 A_82_LPTFS cells, suggesting the virus had evolved in vivo to use a CD46-independent entry mechanism (Figure 4A).

Virus stocks were grown from the original serum samples through three passages on MDBK cells. Both the serum samples and the resulting virus isolates were then sequenced. Surprisingly, despite phenotypic differences observed on *CD46*-edited cells, the consensus genome sequences of the viruses from both serum samples were identical (Figure 4B). However, an examination of the viral quasispecies in the E^RNS^ gene of the adapted virus (PI-86-2022) found there were two nonsynonymous mutations at glycine 479, resulting in arginine (G479R) in 21.2% of reads and lysine (G479K) in 19.1% of reads (Table 2). These mutations are similar to those that arose in vitro after 11 passages on *CD46*-edited cells. Moreover, within the first passage (72 h) of PI-86-2022 serum on either MDBK or MDBK-CD46 A_82_LPTFS cells, the glycine 479 position becomes substituted for a positive charge in over 75% of the reads (Table 2). Specifically, 72 h of infection resulted in the nonsynonymous mutation encoding the G479K substitution being present in 51.4% or 42.6% of reads from the viruses grown on MDBK or MDBK-CD46 A_82_LPTFS cells, respectively. The mutation encoding the G479R substitution was present in 25.7% or 55.5% of reads from the viruses grown on the MDBK cells or MDBK-CD46 A_82_LPTFS cells, respectively. In addition to the glycine 479 substitution, several lower-abundance amino acid substitutions in E^RNS^ were detected in the serum of PI-86-2022 but not PI-86-2021, such as R388Q (32.9%), V434I (23.8%), I480K (21.8%), and Y496H (6.1%) (Supplemental Table S3).

Nonetheless, infections with either the serum samples (Figure 4A) or input viruses (Figure 4C) on MDBK, CD46Δ, and CD46 A_82_LPTFS cells showed that the PI-86-2022 virus was capable of infecting both of the *CD46*-edited cell lines more efficiently compared to the original PI-86-2021 isolate. Heparin pre-treatment (Figure 4D) or removal of surface HS with Heparinase I/III (Figure 4E) significantly impaired the PI-86-2022 virus from infecting the CD46 A_82_LPTFS cells. Thus, the HS-utilizing field strain replicating in a BVDV PI calf displayed similar phenotypes and genotypes as those adapted in vitro on *CD46*-edited MDBK cultures.

### 3.4. Primary Cells from a CD46-Edited Heifer Have Reduced Susceptibility to the In Vitro- and In Vivo-Derived HS-Utilizing Viruses

**Figure 5:**
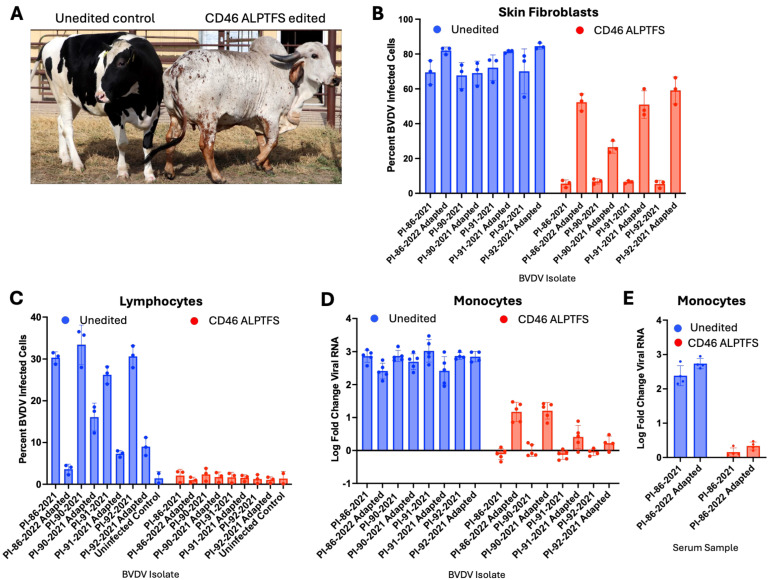
Heparan sulfate-adapted viruses remain restricted in their ability to infect primary cells from a *CD46*-edited heifer. (**A**) Cattle used for primary cell isolation [15]. Primary skin fibroblasts (**B**) or lymphocytes (**C**) were inoculated with unadapted input virus or p11 adapted viruses from MDBK-CD46 A_82_LPTFS cells. BVDV infection was quantified by flow cytometry at 20 hpi. (**D**) Monocytes were inoculated with equal titers of BVDV based on input viral RNA concentrations determined by RT-qPCR. At 48 hpi, viral RNA was detected by RT-qPCR and fold change in viral RNA relative to the input sample (0 hpi) was calculated. Results represent the mean ± SD of three independent experiments. (**E**) Monocytes were inoculated with serum from a BVDV-PI calf and infection was determined by RT-qPCR as described in Panel C.

While these HS-adapted BVDV viruses can readily infect the *CD46*-edited cells in vitro, it was unknown whether these HS-adapted strains were capable of infecting primary cells ex vivo from a *CD46*-edited heifer (Figure 5A). The ability of the unadapted and HS-adapted BVDV strains to infect primary fibroblasts from an unedited and *CD46*-edited heifer was first assessed. All BVDV strains infected unedited skin fibroblasts at comparably high levels (70–80%). However, even though HS-adapted viruses showed some increased infection (30–60%) in *CD46*-edited fibroblasts compared to unadapted viruses (approximately 5%), infectivity in the *CD46*-edited skin fibroblasts was still significantly reduced for all adapted viruses compared to the unedited cells (*p* < 0.002) (Figure 5B).

*CD46*-edited lymphocytes remained highly resistant to both unadapted and HS-adapted viruses (Figure 5C), showing significantly reduced susceptibility compared to unedited cells for most virus isolates (*p* < 0.002, excluding PI-86-2022). This exception, PI-86-2022, displayed inherently low infection in unedited control lymphocytes, potentially precluding the detection of a significant difference. Even when using the maximum volume of each virus for infection, the adapted viruses were highly restricted in their ability to infect lymphocytes from the *CD46*-edited heifer (Supplemental Figure S1). For this maximal virus volume infection, the reduced infectivity of the PI-86-2022 isolate in *CD46*-edited lymphocytes achieved statistical significance (*p* < 0.002). Similar results were seen in monocytes; all virus strains replicated in the unedited cells while only minimal infection of the adapted viruses was detected in monocytes from the *CD46*-edited heifer (Figure 5D). These differences were statistically significant for all virus isolates (*p* < 0.0001). Similarly, monocytes from the *CD46*-edited heifer were resistant to infection by both the unadapted PI-86-2021 and the in vivo HS-adapted PI-86-2022 serum samples (Figure 5E). Taken together, these results indicate that primary cells isolated from a *CD46*-edited heifer remain more resistant to BVDV infection compared to unedited cells, even from viruses adapted to use HS as an alternative entry receptor.

### 3.5. Heparan Sulfate Expression Levels Correlate with Susceptibility to Adapted Viruses

**Figure 6:**
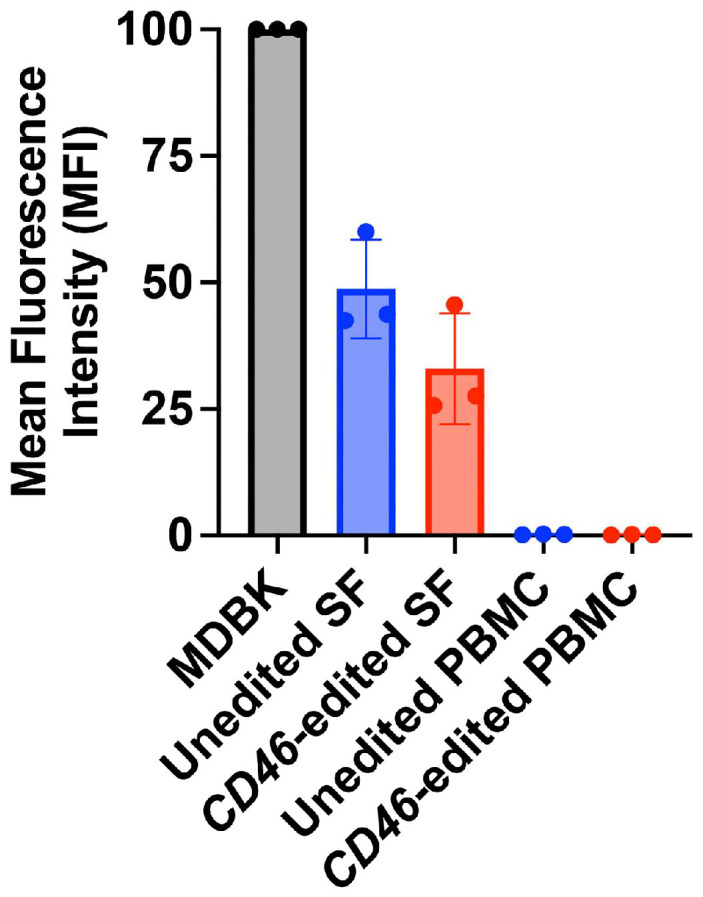
Heparan sulfate surface expression varies between primary skin fibroblasts and PBMCs. Surface heparan sulfate (HS) abundance was quantified by flow cytometry. Results represent the mean ± standard deviation of three independent experiments. SF, skin fibroblast; PBMC, peripheral blood mononuclear cells.

Variations in the levels of HS could potentially explain the differences in susceptibility to HS-adapted BVDV isolates observed between the different types of *CD46*-edited cells. Thus, HS abundance was compared between MDBK cells, peripheral blood mononuclear cells (PBMCs), and primary skin fibroblasts isolated from the unedited or *CD46*-edited heifer. Compared to MDBK cells, primary skin fibroblast cells from the *CD46*-edited heifer had HS levels reduced by approximately three-fold, and those from the unedited heifer had levels reduced by two-fold (Figure 6). HS detection in PBMCs from both unedited and *CD46*-edited heifers was minimal compared to MDBK cells. Thus, HS expression levels strongly correlate with cell susceptibility to the adapted viruses, further supporting the hypothesis that the adapted viruses are using a HS-dependent entry mechanism to infect *CD46*-edited cells.

## 4. Discussion

Evaluating viral adaptation to host genetic modifications is crucial for assessing the long-term efficacy of gene-editing strategies in disease control. The present study demonstrates that in vitro BVDV adaptation to infect *CD46*-edited MDBK cells occurs via a CD46-independent mechanism involving increased use of HS as an alternative entry receptor. However, these HS-adapted viruses, including a field isolate from a BVDV PI calf, exhibited restricted tropism in primary cells from a *CD46*-edited heifer. These results underscore the complexity of viral adaptation and the importance of evaluating gene-editing strategies in diverse cellular contexts.

HS adaptation is a frequent consequence of in vitro virus propagation observed across diverse viral families, including several *Flaviviridae* members [20,21,24,25,26,27,28,29]. Notably, BVDV is known to acquire HS-adaptive mutations in vitro to overcome infection restriction in MDBK cells lacking CD46 [10,14]. A G479R substitution in the BVDV surface glycoprotein E^RNS^ is predicted to increase HS-binding affinity due to the net gain of positive charge [6,20,30]. Two of the three in vitro HS-adapted viruses in this study (PI-91-2021 and PI-92-2021) contain the same G479R substitution in E^RNS^. Furthermore, the in vivo-derived HS-adapted serum isolate PI-86-2022 from a PI yearling contained an analogous, positively charged residue at this position: either lysine (19.1%) or arginine (21.2%). Although these HS-adaptive amino acids did not reach the 50% threshold for inclusion in the consensus genome sequence, their presence at lower frequencies in the viral quasispecies was sufficient to confer the adaptive phenotype in vitro. The third in vitro-adapted isolate (PI-90-2021) lacked known HS-adaptive substitutions in its E^RNS^ gene. However, E^RNS^ quasispecies analysis identified several candidate mutations requiring further investigation. Nevertheless, given the high level of HS expression in MDBK cells, rapid HS adaptation likely minimized the selection pressure for BVDV to use the gene-edited CD46 A_82_LPTFS receptor to initiate infection.

While in vitro HS adaptation can provide a selective advantage in cell culture, it can also lead to trade-offs that reduce viral fitness in vivo. Indeed, in vitro-derived HS-adaptive mutations in other *Flaviviridae* members have been associated with reduced in vivo virulence [21,25,26,31]. Consistent with this, the ex vivo studies here showed that white blood cells from the *CD46*-edited heifer expressed low levels of HS and remained highly resistant to HS-adapted BVDV variants. Given the crucial role of white blood cells in systemic BVDV dissemination, these data suggest that HS-adapted variants may have an attenuated phenotype in vivo. In BVDV, enhanced E^RNS^ affinity for HS may also impact in vivo pathogenesis by altering the entry mechanism involving the primary receptor, CD46. This is supported by the results demonstrating the marked reduction in infection of heparin- or Heparinase I/III-treated MDBK cells by adapted, but not unadapted, BVDV isolates. Similar results have been reported for HS-adapted CSFV [20,32] and *Pestivirus* H [13]. Although the underlying molecular mechanisms remain to be fully elucidated, this observation could be explained by two models. In the first model, in the absence of sufficient HS levels to warrant infection, decreased use of its primary receptor would negatively impact infection. In a second model, HS may not be sufficient for virus entry in some cell types in vivo. Therefore, increased affinity for HS may sequester the virus away from CD46 or other yet undiscovered entry receptors and thus prevent infection. Thus, the in vivo impact of the interplay between HS and CD46 use on BVDV pathogenesis and transmission will require further investigation in both unedited and *CD46*-edited cattle.

A question that remains is how well in vitro viral evolution studies predict the adaptations that may arise in gene-edited livestock. While in vitro models provide valuable insights into potential viral escape mechanisms, they have limitations in replicating the complex selective pressures of the in vivo environment. For example, in vitro studies often focus on viral growth and replication within a single cell type, whereas in vivo infections involve diverse cell types, host immune responses, and transmission dynamics. The challenge in predicting viral adaptations that may arise in vivo is further compounded by the presence of viral quasispecies, whose genetic diversity contributes to the virus’s ability to adapt to diverse environments. Thus, predicting how viral quasispecies will respond to the diverse selective pressures within a gene-edited host remains a key challenge for in vitro approaches evaluating the potential durability of genetic modifications.

The case of BVDV in PI cattle provides an example of diverse selective pressures on viral evolution and highlights the challenges in predicting viral adaptation. Because PI cattle are established in utero before immune competence, the calf remains immunotolerant to the infecting virus. The absence of adaptive immune pressure in PI cattle permits high levels of BVDV replication in a wide range of tissues [4]. This extensive replication increases the opportunity for new mutations to arise and for the virus to adapt to different cellular environments, resulting in a more diverse viral quasispecies [33,34]. Consequently, while the detection of an in vivo-derived HS-utilizing virus in a PI yearling is noteworthy, it may not accurately reflect the likelihood of such adaptations arising de novo during acute infections in *CD46*-edited livestock, where viral replication is substantially restricted [12]. In the event a *CD46*-edited bovine is exposed to a virus population containing viral variants capable of CD46-independent entry, this new selective pressure could favor these viral variants. This selection of already-present adaptive variants, rather than the emergence of de novo mutations, is a mechanism of viral adaptation to consider. However, the frequency at which these HS-adapted viruses arise in PI cattle, their transmission potential, and their impact on disease phenotypes remain unknown. Understanding these viral adaptation mechanisms in vivo will help guide the development of durable BVDV control strategies.

## 5. Conclusions

The edited CD46 A_82_LPTFS receptor eliminates CD46-dependent virus entry. This creates selective pressure for viral variants to emerge capable of either using an alternative entry pathway or adapting to the edited receptor. Our in vitro serial passage model demonstrated that BVDV can rapidly evolve a CD46-independent mechanism of entry involving increased use of HS as an alternative receptor. However, it remains unknown whether the distinct selective pressures present in vivo would also select for HS-adapted BVDV viruses in *CD46*-edited cattle, and if so, how BVDV tropism, pathogenesis, and disease outcomes would be impacted.

## Figures and Tables

**Table 1:** Targeted viral quasispecies analysis of E^RNS^ for in vitro adapted viruses. (**Top**) Accession numbers for the consensus sequence of the indicated virus isolates and the average sequence read depth across the viral genome. (**Bottom**) Amino acid substitutions in the viral glycoprotein E^RNS^ that have been characterized in prior studies [7,14,20,21,22,23]. Sites that are marked with an asterisk (*) indicate no other amino acids are detected at a frequency above 0.5%. Grey highlight denotes table headers.

	PI-90-21	Serum	INPUT	p11 MDBK	p11 CD46Δ	p11 CD46 ALPTFS
	Accession	PQ613788	PQ613786	PQ613787	PQ613785	PQ613784
	Read Depth	1374	144,875	75,534	116,976	130,942
	PI-91-21	Serum	INPUT	p11 MDBK	p11 CD46Δ	p11 CD46 ALPTFS
	Accession	PQ613793	PQ613791	PQ613792	PQ613790	PQ613789
	Read depth	831	162,529	54,832	83,482	126,951
	PI-92-21	Serum	INPUT	p11 MDBK	p11 CD46Δ	p11 CD46 ALPTFS
	Accession	PQ613798	PQ613796	PQ613797	PQ613795	PQ613794
	Read depth	559	210,742	84,011	89,169	159,937
Amino acid, position	Virus strain	Serum	INPUT	p11 MDBK	p11 CD46Δ	p11 CD46 ALPTFS
	PI-90-21	*	*	*	*	*
Histidine, 300	PI-91-21	Leucine (1.6%)	*	*	*	*
	PI-92-21	Leucine (0.7%)	*	Arginine (23%)	Arginine (83.0%)	Arginine (81.7%)
	PI-90-21	*	*	*	*	*
Cysteine, 441	PI-91-21	*	*	*	*	*
	PI-92-21	*	*	*	*	*
	PI-90-21	*	*	*	*	*
Glycine, 479	PI-91-21	Arginine (2%)	*	Arginine (43.5%)	Arginine (94.9%)	Arginine (90.2%)
	PI-92-21	*	*	Arginine (25.8%)	Arginine (98.1%)	Arginine (98.0%)
	PI-90-21	Valine (1.7%)	Valine (0.8%)	Threonine (0.5%)	Valine (0.5%)	*
		Leucine (0.5%)				
Isoleucine, 480	PI-91-21	Lysine (1.8%)	*	Lysine (27.8%)	Lysine (76.1%)	Lysine (69.8%)
		Arginine (0.9%)		Arginine (15.8%)	Arginine (19.2%)	Arginine (21.1%)
	PI-92-21	Threonine (0.9%)	*	Lysine (25%)	Lysine (82.7%)	Lysine (83.8%)
		Leucine (0.5%)				

**Table 2:** Targeted viral quasispecies analysis of E^RNS^ for in vivo adapted viruses. (**Top**) Accession numbers for the consensus sequence of the indicated virus isolates and the average sequence read depth across the viral genome. (**Bottom**) Amino acid substitutions in the viral glycoprotein E^RNS^ that have been characterized in prior studies [7,14,20,21,22,23]. Sites that are marked with an asterisk (*) indicate no other amino acids are detected at a frequency above 0.5%. Grey highlight denotes table headers.

	PI-86-21	Serum	72 h MDBK	72 h MDBK A_82_LPTFS	INPUT
	Accession	PQ613779			PQ613778
	Read Depth	1967			2361
	PI-86-22	Serum	72 h MDBK	72 h MDBK A_82_LPTFS	INPUT
	Accession	PQ613783	PQ613781	PQ613780	PQ613782
	Read depth	5377	108,859	206,498	1060
Amino acid, position	Virus strain	Serum	72 h MDBK	72 h MDBK A_82_LPTFS	INPUT
	PI-86-21	*			*
Histidine, 300	PI-86-22	Tyrosine (0.5%)	Arginine (0.5%)	Tyrosine (0.7%)	*
				Arginine (0.6%)	
				Leucine (0.6%)	
	PI-86-21	*			*
Cysteine, 441	PI-86-22	*	*	*	*
	PI-86-21	Arginine (2.6%)			Arginine (1%)
Glycine, 479	P8-86-22	Arginine (21.2%)	Lysine (51.4%)	Arginine (55.5%)	Lysine (79.6%)
		Lysine (19.1%)	Arginine (25.7%)	Lysine (42.6%)	Arginine (11.2%)
	PI-86-21	Lysine (1.9%)			*
		Arginine (0.6%)			
		Threonine (0.5%)			
Isoleucine, 480	PI-86-22	Lysine (21.8%)	Lysine (24%)	Lysine (58.1%)	Lysine (11.1%)
		Methionine (2.5%)	Methionine (7.4%)	Methionine (8.3%)	
		Valine (1.0%)	Threonine (3.6%)	Valine (1.8%)	
		Threonine (0.7%)		Arginine (0.5%)	
		Arginine (0.6%)			

## Data Availability

BVDV consensus genomes were submitted to Genbank under accession numbers PQ613778-PQ613798.

## Supplementary Materials

The following supporting information can be downloaded at: https://www.mdpi.com/article/10.3390/v17050634/s1, Figure S1: Infection of lymphocytes with maximal amounts of unadapted and adapted virus pairs; Table S1: Nucleotide changes acquired during serial passage of noncytopathic BVDV isolates; Table S2: Viral quasispecies analysis of E^RNS^ for in vitro adapted viruses; Table S3: Viral quasispecies analysis of E^RNS^ for in vivo adapted PI-86-22.

## Abbreviations

The following abbreviations are used in this manuscript:

BVDV	Bovine Viral Diarrhea Virus
CRISPRs	Clustered Regularly Interspaced Short Palindromic Repeats
HS	Heparan Sulfate
MDBK	Madin–Darby Bovine Kidney
PBMC	Peripheral Blood Mononuclear Cell
PI	Persistently Infected
