# Supplementary material for: Primary Cells from a CD46-Edited Bovine Heifer Have Reduced BVDV Susceptibility Despite Viral Adaptation to Heparan Sulfate
Source: Viruses. 2025 Apr 28;17(5):634. doi: 10.3390/v17050634 (PMC12116123; doi:10.3390/v17050634)
Supplement: Supplementary file 1 [file viruses-17-00634-s001.zip › Supplemental Figure S1.pdf]

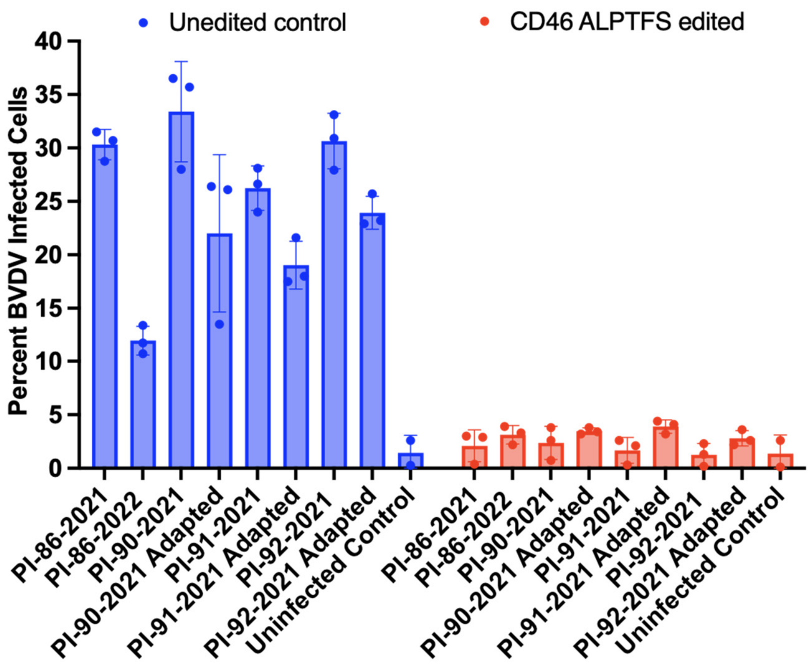

Supplemental Figure S1. Infection of lymphocytes with maximal amounts of unadapted and adapted virus pairs. Given the low infection rates observed with the adapted viruses, we further increased the viral titer by adding the maximal volume of 80 uL per well. Thus, lymphocytes were inoculated with 80 uL unadapted input virus (average MOI =5; same data as Figure 5) or p11 adapted viruses from CD46 $A_{82}LPTFS_{87}$ cells (average MOI =27). BVDV infection was quantified by flow cytometry at 20 hpi.
