# Supplementary material for: Primary Cells from a CD46-Edited Bovine Heifer Have Reduced BVDV Susceptibility Despite Viral Adaptation to Heparan Sulfate
Source: Viruses. 2025 Apr 28;17(5):634. doi: 10.3390/v17050634 (PMC12116123; doi:10.3390/v17050634)
Supplement: Supplementary file 1 [file viruses-17-00634-s001.zip › Supplemental Table S2.pdf]

Amino acid, position	Virus strain	Serum	INPUT	p11 MDBK	p11 CD46Δ	p11 CD46 ALPTFS
Asparagine, 277	PI-90-21	Aspartic acid (0.5%)<br>Serine (0.5%)	Serine (1.5%)	Serine (1.2%)	Serine (0.9%)	Serine (1%)
	PI-91-21	*	Serine (0.6%)	*	*	*
	PI-92-21	Serine (0.5%)	*	*	*	*
Isoleucine, 286	PI-90-21	Valine (0.5%)	Arginine (0.6%)	Threonine (0.8%)	Threonine (0.5%)	Threonine (0.7%)
	PI-91-21	*	*	*	*	*
	PI-92-21	Valine (0.5%)	Valine (0.6%)	Valine (0.5%)	*	*
Valine, 295	PI-90-21	Alanine (1.8%)<br>Glycine (0.6%)	Glycine (1.5%)<br>Glutamic acid (0.7%)<br>Alanine (0.7%)	Alanine (1.1%)<br>Glycine (0.6%)<br>Glutamic acid (0.5%)	Alanine (0.6%)	Alanine (1.1%)<br>Glycine (0.8%)
	PI-91-21	*	*	*	*	*
	PI-92-21	*	*	*	*	*
**Histidine, 300**	PI-90-21	*	*	*	*	*
	PI-91-21	Leucine (1.6%)	*	*	*	*
	PI-92-21	Leucine (0.7%)	*	Arginine (23%)	Arginine (83.0%)	Arginine (81.7%)
Proline, 304	PI-90-21	*	Serine (1.3%)	*	Serine (0.6%)	Serine (0.9%)
	PI-91-21	*	*	*	*	*
	PI-92-21	Serine (0.7%)	*	*	*	*
Tyrosine, 355	PI-90-21	Cysteine (1.7%)<br>Histidine (0.7%)<br>Asparagine (0.6%)<br>Phenylalanine (0.5%)	Cysteine (0.9%)<br>Phenylalanine (0.7%)<br>Asparagine (0.6%)<br>Histidine (0.5%)	Histidine (0.6%)<br>Phenylalanine (0.5%)<br>Cysteine (0.5%)	Histidine (0.8%)<br>Cysteine (0.7%)<br>Arginine (0.5%)	Cysteine (0.7%)<br>Asparagine (0.6%)<br>Isoleucine (0.5%)
	PI-91-21	Phenylalanine (0.7%)	Cysteine (0.6%)<br>Asparagine (0.5%)	*	Histidine (0.8%)<br>Phenylalanine (0.7%)<br>Cysteine (0.7%)	*
	PI-92-21	Histidine (0.6%)	*	*	Histidine (0.5%)	Histidine (0.5%)
Aspartic Acid, 389	PI-90-21	Glycine (1.9%)	Glycine (1.8%)	Glycine (1.4%)<br>Alanine (0.6%)	*	Glycine (1.5%)
	PI-91-21	*	*	*	*	*
	PI-92-21	*	*	*	*	*
Glycine, 411	PI-90-21	Glutamic acid (1.9%)	Glutamic acid (1.5%)	Glutamic acid (0.9%)	Glutamic acid (1.2%)	Glutamic acid (1.3%)
	PI-91-21	*	*	*	*	*
	PI-92-21	*	Aspartic acid (0.7%)	*	*	*
Lysine, 412	PI-90-21	Glutamic acid (1.3%)	Glutamic acid (4%)<br>Stop codon (1.6%)<br>Arginine (0.9%)<br>Glutamine (0.7%)<br>Isoleucine (0.5%)	Glutamine (0.8%)<br>Stop codon (0.7%)<br>Glutamic acid (0.7%)	Glutamic acid (1.1%)<br>Stop codon (0.5%)<br>Glutamine (0.5%)	Glutamic acid (1%)<br>Glutamine (0.6%)<br>Stop codon (0.5%)
	PI-91-21	Glutamine (0.8%)	Stop codon (0.9%)<br>Glutamine (0.5%)	Glutamine (0.8%)<br>Stop codon (0.7%)	Stop codon (0.5%)<br>Glutamic acid (0.5%)<br>Glutamine (0.5%)	Glutamic acid (1.3%)<br>Stop codon (1.1%)<br>Glutamine (0.8%)
	PI-92-21	Glutamic acid (0.7%)	Glutamic acid (1.2%)<br>Glutamine (0.7%)<br>Stop codon (0.5%)	Glutamine (0.6%)	Glutamine (0.5%)	Glutamine (0.6%)
Methionine, 421	PI-90-21	*	*	Arginine (11.8%)	Arginine (28.9%)	Arginine (34.9%)
	PI-91-21	Valine (99.9%)	Valine (99.8%)	Valine (99.9%)	Valine (99.8%)	Valine (99.8%)
	PI-92-21	Valine (99.4%)<br>Alanine (0.6%)	Valine (99.8%)	Valine (99.7%)	Valine (99.8%)	Valine (99.7%)

Arginine, 422	PI-90-21	Glutamine (1.1%)	Glutamine (1.2%)<br>Tryptophan (0.6%)	Glutamine (0.8%)<br>Tryptophan (0.5%)	Glutamine (0.8%)	Tyrosine (0.8%)
	PI-91-21	Glutamine (99.8%)	Glutamine (99.8%)	Glutamine (99.8%)	Glutamine (99.7%)	Glutamine (99.7%)
	PI-92-21	Glutamine (98.1%)<br>Leucine (1.9%)	Glutamine (99.7%)	Glutamine (99.8%)	Glutamine (99.7%)	Glutamine (99.8%)
Glycine, 423	PI-90-21	Aspartic acid (0.9%)<br>Serine (0.5%)	Serine (0.6%)	Valine (0.6%)<br>Aspartic acid (0.5%)	Aspartic acid (0.5%)	Serine (0.9%)
	PI-91-21	*	*	*	*	*
	PI-92-21	*	*	*	*	*
Aspartic Acid, 430	PI-90-21	Alanine (3.0%)<br>Glycine (0.5%)	Alanine (1.7%)	Alanine (11.3%)	Alanine (26.9%)<br>Valine (3.5%)<br>Tyrosine (1.3%)	Alanine (33.1%)<br>Tyrosine (8%)<br>Valine (4.1%)
	PI-91-21	Alanine (99.1%)<br>Threonine (0.5%)	Alanine (99.7%)	Alanine (99.8%)	Alanine (99.8%)	Alanine (99.8%)
	PI-92-21	Alanine (98.0%)<br>Valine (2.0%)	Alanine (98.7%)<br>Threonine (1.2%)	Alanine (99.4%)<br>Threonine (0.5%)	Alanine (99.8%)	Alanine (99.8%)
**Cysteine, 441**	PI-90-21	*	*	*	*	*
	PI-91-21	*	*	*	*	*
	PI-92-21	*	*	*	*	*
Methionine, 456	PI-90-21	Arginine (1.1%)<br>Threonine (0.6%)	Arginine (2.7%)<br>Threonine (0.7%)	Valine (11.8%)	Valine (26.5%)	Valine (36.9%)
	PI-91-21	Threonine (1.4%)<br>Valine (0.8%)	Arginine (3.4%)<br>Threonine (0.9%)<br>Valine (0.6%)	Threonine (0.8%)<br>Arginine (0.5%)	Threonine (1.3%)<br>Arginine (0.7%)	Arginine (0.9%)
	PI-92-21	Arginine (1.9%)<br>Threonine (0.8%)	Arginine (2%)<br>Threonine (0.5%)	Threonine (0.8%)<br>Arginine (0.7%)	Threonine (0.7%)<br>Arginine (0.6%)	Threonine (0.5%)<br>Arginine (0.5%)
**Glycine, 479**	PI-90-21	*	*	*	*	*
	PI-91-21	Arginine (2%)	*	Arginine (43.5%)	Arginine (94.9%)	Arginine (90.2%)
	PI-92-21	*	*	Arginine (25.8%)	Arginine (98.1%)	Arginine (98.0%)
**Isoleucine, 480**	PI-90-21	Valine (1.7%)<br>Leucine (0.5%)	Valine (0.8%)	Threonine (0.5%)	Valine (0.5%)	*
	PI-91-21	Lysine (1.8%)<br>Arginine (0.9%)	*	Lysine (27.8%)<br>Arginine (15.8%)	Lysine (76.1%)<br>Arginine (19.2%)	Lysine (69.8%)<br>Arginine (21.1%)
	PI-92-21	Threonine (0.9%)<br>Leucine (0.5%)	*	Lysine (25%)	Lysine (82.7%)	Lysine (83.8%)
Glycine, 482	PI-90-21	Glutamic acid (1.5%)	Glutamic acid (1.4%)	Glutamic acid (0.9%)	Glutamic acid (1%)	Glutamic acid (1.5%)
	PI-91-21	*	*	*	*	*
	PI-92-21	*	*	*	*	*

Supplemental Table S2. Viral quasispecies analysis of E^{RNS} for in vitro adapted viruses. Overview of all the amino acid substitutions in the viral glycoprotein E^{rns} with an emphasis on those that are above 0.5% and occur in PI-90-21. Substitutions that have occurred in prior studies (also included in Table 1) are bolded and underlined. Sites that are marked with (*) indicate no other amino acids are detected at a frequency above 0.5%.
