# Supplementary material for: Primary Cells from a CD46-Edited Bovine Heifer Have Reduced BVDV Susceptibility Despite Viral Adaptation to Heparan Sulfate
Source: Viruses. 2025 Apr 28;17(5):634. doi: 10.3390/v17050634 (PMC12116123; doi:10.3390/v17050634)
Supplement: Supplementary file 1 [file viruses-17-00634-s001.zip › Supplemental Table S3.pdf]

Amino acid, position	Virus strain	Serum	72hr MDBK	72hr MDBK-A_{82}LPTFS	INPUT
Isoleucine, 273	PI-86-21	*			*
	PI-86-22	Threonine (1.4%)<br>Lysine (0.5%)	Lysine (0.7%)	Lysine (0.5%)	*
**Histidine, 300**	PI-86-21	*			*
	PI-86-22	Tyrosine (0.5%)	Arginine (0.5%)	Tyrosine (0.7%)<br>Arginine (0.6%)<br>Leucine (0.6%)	*
Proline, 304	PI-86-21	*			*
	PI-86-22	Serine (0.7%)<br>Leucine (0.5%)	Serine (0.8%)<br>Leucine (0.6%)<br>Threonine (0.5%)	Leucine (0.7%)<br>Threonine (0.6%)<br>Serine (0.5%)	*
Glutamic acid, 305	PI-86-21	Lysine (0.5%)			*
	PI-86-22	Alanine (0.8%)<br>Valine (0.5%)	Stop codon (0.5%)	Stop codon (0.7%)<br>Valine (0.5%)	*
Lysine, 306	PI-86-21	Glutamic acid (0.6%)			*
	PI-86-22	Glutamic acid (1.4%)<br>Stop codon (0.5%)	Glutamic acid (0.7%)<br>Stop codon (0.6%)	Glutamic acid (0.8%)<br>Stop codon (0.5%)<br>Glutamine (0.5%)	*
Valine, 311	PI-86-21	Isoleucine (5.5%)			*
	PI-86-22	Isoleucine (1.2%)	*	Isoleucine (0.8%)	*
Threonine, 319	PI-86-21	*			*
	PI-86-22	Isoleucine (4.4%)	*	*	Isoleucine (1.5%)
Lysine, 322	PI-86-21	*			*
	PI-86-22	Glutamine (1.1%)	Glutamine (0.6%)	Glutamine (3.9%)	Glutamine (1.2%)
Methionine, 328	PI-86-21	*			*
	PI-86-22	Leucine (2.5%)	Leucine (0.7%)	Leucine (0.7%)	*
Lysine, 333	PI-86-21	Methionine (0.8%)			Glutamic acid (0.6%)<br>Arginine (0.8%)
	PI-86-22	Methionine (1.8%)<br>Glutamic acid (1.4%)<br>Arginine (1.4%)<br>Stop codon (0.9%)	Glutamic acid (1.1%)<br>Arginine (1.1%)<br>Stop codon (0.7%)<br>Methionine (0.6%)	Arginine (1.4%)<br>Glutamic acid (1.3%)<br>Stop codon (0.8%)<br>Methionine (0.6%)	Glutamic acid (1.2%)<br>Glutamine (0.5%)
Threonine, 334	PI-86-21	Alanine (0.5%)			Serine (0.8%)
	PI-86-22	Alanine (1.1%)<br>Arginine (1.0%)<br>Serine (0.55)	Serine (0.9%)<br>Arginine (0.9%)<br>Alanine (0.6%)<br>Lysine (0.5%)	Arginine (1.3%)<br>Serine (0.9%)<br>Alanine (0.6%)<br>Lysine (0.5%)	*
Asparagine, 347	PI-86-21	*			Arginine (0.5%)
	PI-86-22	Aspartic acid (0.8%)<br>Serine (0.7%)	Serine (0.5%)	Isoleucine (0.6%)<br>Serine (0.6%)<br>Aspartic acid (0.5%)	Serine (0.6%)
Histidine, 349	PI-86-21	Serine (1.0%)			*
	PI-86-22	Arginine (0.8%)	*	*	*
Tryptophan, 351	PI-86-21	*			*
	PI-86-22	Glycine (1.1%)<br>Arginine (0.5%)	Arginine (0.5%)	Arginine (0.5%)<br>Cysteine (0.5%)	*<br>*
Tyrosine, 355	PI-86-21	*			*
	PI-86-22	Histidine (0.8%)	Histidine (1.5%)	*	Histidine (0.8%)

Proline, 359	PI-86-21	Serine (1.2%)			*
	PI-86-22	Serine (1.1%)<br>Threonine (1.1%)	*	Leucine (0.6%)<br>Arginine (0.5%)	Serine (2.5%)
Arginine, 388	PI-86-21	*			*
	PI-86-22	Glutamine (32.9%)	Glutamine (1.2%)	*	Glutamine (1.3%)
Aspartic acid, 400	PI-86-21	*			*
	PI-86-22	Asparagine (1.0%)	Alanine (0.6%)	*	*
Valine, 434	PI-86-21	*			*
	PI-86-22	Isoleucine (23.8%)	Isoleucine (8.0%)	*	*
<u>**Cysteine, 441**</u>	PI-86-21	*			*
	PI-86-22	*	*	*	*
Tyrosine, 451	PI-86-21	*			*
	PI-86-22	Histidine (1.1%)	Histidine (0.7%)	Histidine (2.8%)	*
Methionine, 456	PI-86-21	Cysteine (4.65%)<br>Valine (0.5%)			Arginine (0.8%)
	PI-86-22	Arginine (1.2%)<br>Valine (0.8%)	Arginine (1.2%)<br>Valine (0.7%)<br>Leucine (0.6%)<br>Lysine (0.6%)<br>Threonine (0.6%)	Isoleucine (0.6%)	Arginine (0.8%)
Leucine, 474	PI-86-21	Phenylalanine (0.6%)			*
	PI-86-22	Phenylalanine (1.4%)	Phenylalanine (4.9%)	Phenylalanine (5.4%)	Phenylalanine (2.0%)
Leucine 478	PI-86-21	*			*
	PI-86-22	Phenylalanine (2.5%)<br>Proline (0.7%)	Phenylalanine (2.4%)	Phenylalanine (8.9%)	Phenylalanine (2.7%)
<u>**Glycine, 479**</u>	PI-86-21	Arginine (2.6%)			Arginine (1%)
	PI-86-22	Arginine (21.2%)<br>Lysine (19.1%)	Lysine (51.4%)<br>Arginine (25.7%)	Arginine (55.5%)<br>Lysine (42.6%)	Lysine (79.6%)<br>Arginine (11.2%)
<u>**Isoleucine, 480**</u>	PI-86-21	Lysine (1.9%)<br>Arginine (0.6%)<br>Threonine (0.5%)			*
	PI-86-22	Lysine (21.8%)<br>Methionine (2.5%)<br>Valine (1.0%)<br>Threonine (0.7%)<br>Arginine (0.6%)	Lysine (24%)<br>Methionine (7.4%)<br>Threonine (3.6%)	Lysine (58.1%)<br>Methionine (8.3%)<br>Valine (1.8%)<br>Arginine (0.5%)	Lysine (11.1%)
Glutamic acid, 486	PI-86-21	Glycine (0.5%)			*
	PI-86-22	Glycine (0.6%)	Glycine (0.8%)	Glycine (0.7%)	Glycine (0.8%)
Glycine, 494	PI-86-21	Arginine (0.6%)<br>Glutamic acid (0.6%)			Arginine (0.9%)
	PI-86-22	Arginine (0.5%)	Arginine (0.5%)	Arginine (1.1%)<br>Glutamic acid (0.5%)<br>Valine (0.5%)	Glutamic acid (0.6%)
Tyrosine, 496	PI-86-21	*			*
	PI-86-22	Histidine (6.1%)	Histidine (8.2%)	Histidine (19.2%)<br>Aspartate (0.7%)<br>Aspartic acid (0.7%)<br>Phenylalanine (0.5%)	Histidine (3.3%)

Supplemental Table S3. Viral quasispecies analysis of E^{RNS} for in vivo adapted PI-86-22. Overview of all the amino acid substitutions in the viral glycoprotein E^{rns} with an emphasis on those that are above 0.5% and occur in the PI-86-22 serum sample. Substitutions that have occurred in prior studies (also included in Table 2) are bolded and underlined. Sites that are marked with (*) indicate no other amino acids are detected at a frequency above 0.5%.
